# Supplementary figures and images for: The Dps4 from Nostoc punctiforme ATCC 29133 is a member of His-type FOC containing Dps protein class that can be broadly found among cyanobacteria
Source: PLoS One. 2019 Aug 1;14(8):e0218300. doi: 10.1371/journal.pone.0218300 (PMC6675082; doi:10.1371/journal.pone.0218300)

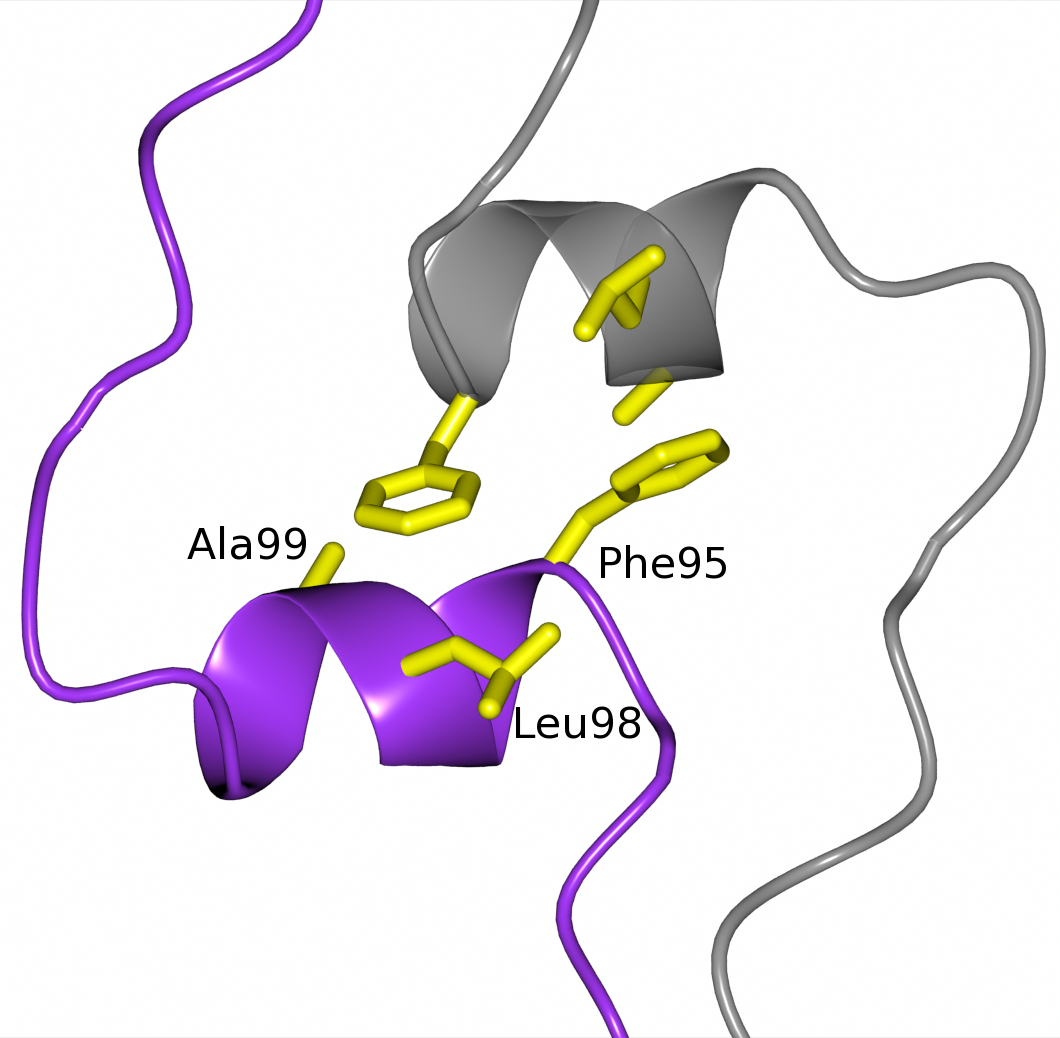

Supplement: S1 Fig — Hydrophobic interaction between the BC helix from two subunits depicted in grey and purple, respectively. Residues Phe95, Leu98 and Ala99 are depicted as stick models. (TIFF) [file pone.0218300.s001.tiff]

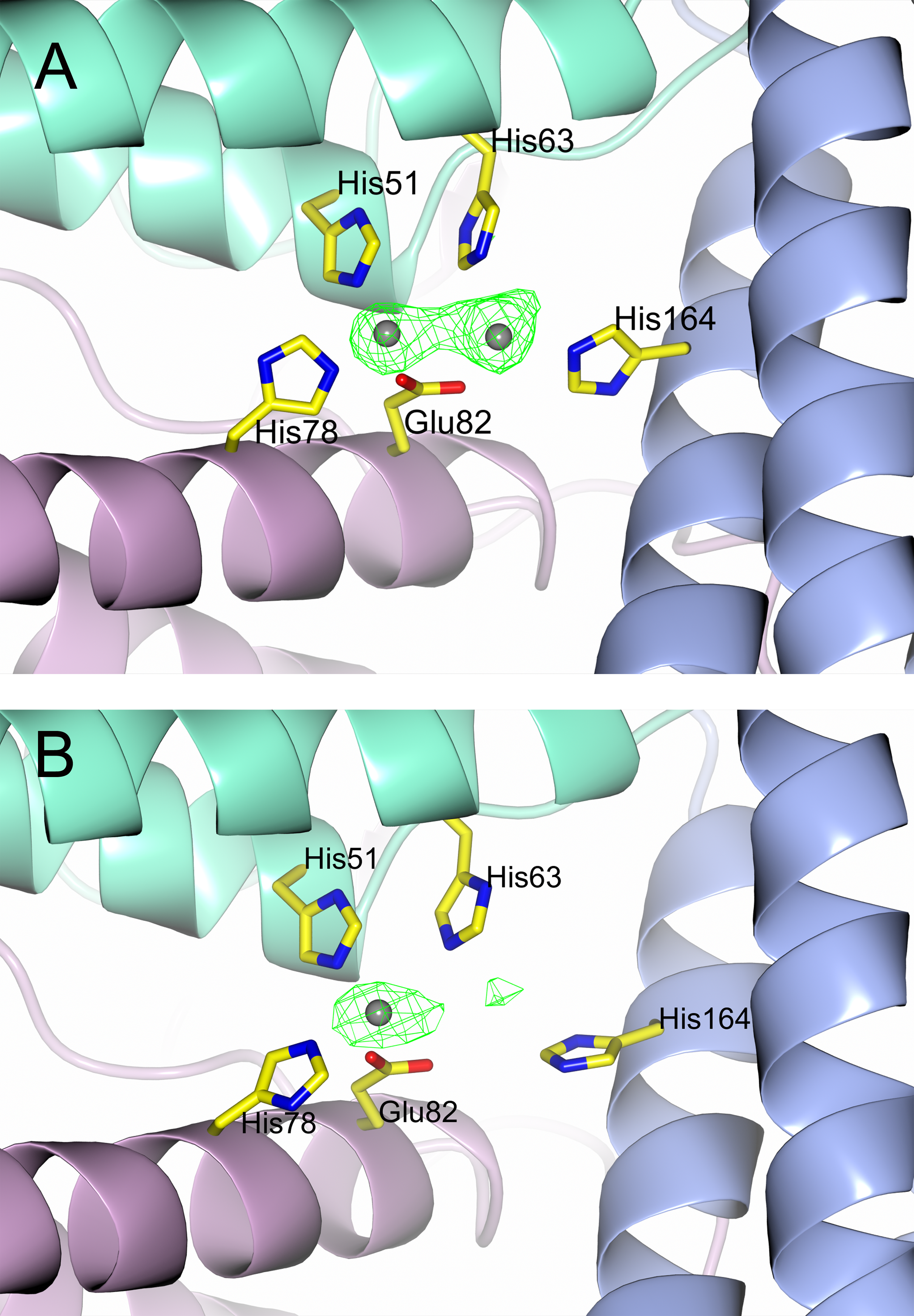

Supplement: S2 Fig — An anomalous difference map (3σ) in green indicates the position of the metals. A. The anomalous density confirms two metals bound (A- and B-site) in the Fe-soaked protein. B. In the Zn-soaked protein the anomalous density indicate one metal with high occupancy bound in the A-site. The density also suggests the possibility of a Zn-atom in the B-site, however this metal has not been modelled due to low occupancy. (TIF) [file pone.0218300.s002.tif]
