# Supplementary material for: The Dps4 from Nostoc punctiforme ATCC 29133 is a member of His-type FOC containing Dps protein class that can be broadly found among cyanobacteria
Source: PLoS One. 2019 Aug 1;14(8):e0218300. doi: 10.1371/journal.pone.0218300 (PMC6675082; doi:10.1371/journal.pone.0218300)
Supplement: S1 Table — The coordination distances of the A and B sites. (DOCX) [file pone.0218300.s004.docx]

| FOC site 1 | | | FOC site 2 | | | FOC site 3 | | | FOC site 4 | | |
| --- | --- | --- | --- | --- | --- | --- | --- | --- | --- | --- | --- |
| Metal site A | | | Metal site A | | | Metal site A | | | Metal site A | | |
|  | Distance (Å) | |  | Distance (Å) | |  | Distance (Å) | |  | Distance (Å) | |
| Coordinating residue | Fe | Zn | Coordinating residue | Fe | Zn | Coordinating residue | Fe | Zn | Coordinating residue | Fe | Zn |
| His78(A) | 2.29 | 2.32 | His78(B) | 2.02 | 2.33 | His78(D) | 2.01 | 2.17 | His78(D) | 2.21 | 2.23 |
| Glu82(A) | 1.84 | 2.46 | Glu82(A) | 1.94 | 2.15 | Glu82(C) | 2.40/  3.18 | 2.64 | Glu82(D) | 2.60/  3.08 | 3.23  /3.35 |
| His51(B) | 2.44 | 2.42 | His51(A) | 2.32 | 2.40 | His51(D) | 2.37 | 2.45 | His51(C) | 2.47 | 2.43 |
|  |  |  |  |  |  |  |  |  |  |  |  |
| FOC site 1 | | | FOC site 2 | | | FOC site 3 | | | FOC site 4 | | |
| Metal site B | | | Metal site B | | | Metal site B | | | Metal site B | | |
|  | Fe | |  | Fe | |  | Fe | |  | Fe | |
| Coordinating residue |  | | Coordinating residue |  | | Coordinating residue |  | | Coordinating residue |  | |
| His63(B) | 2.38 | | His63(A) | 2.65 | | His63(D) | 2.38 | | His63(C) | 2.33 | |
| Glu82(A) | 3.16/2.91 | | Glu82(B) | 2.64 | | Glu82(C) | 2.47 | | Glu82(D) | 2.79 | |
| His164 (symm) | 2.80 | | His164 (symm) | 2.42 | | His164 (symm) | 3.71 | | His164 (symm) | 2.81 | |
|  |  | |  |  | |  |  | |  |  | |
