# Supplementary material for: The Dps4 from Nostoc punctiforme ATCC 29133 is a member of His-type FOC containing Dps protein class that can be broadly found among cyanobacteria
Source: PLoS One. 2019 Aug 1;14(8):e0218300. doi: 10.1371/journal.pone.0218300 (PMC6675082; doi:10.1371/journal.pone.0218300)
Supplement: S2 File — Full wwPDB X-ray structure validation report for 5hjf. (PDF) [file pone.0218300.s006.pdf]

Full wwPDB X-ray Structure Validation Report ⓘ

Mar 9, 2018 – 11:12 pm GMT

PDB ID	:	5HJF
Title	:	The apo form of Dps4 from Nostoc punctiforme
Authors	:	Howe, C.; Moparthi, V.K.; Persson, K.; Stensjo, K.
Deposited on	:	2016-01-13
Resolution	:	1.59 Å(reported)

This is a Full wwPDB X-ray Structure Validation Report for a publicly released PDB entry.

We welcome your comments at *validation@mail.wwpdb.org*
A user guide is available at
https://www.wwpdb.org/validation/2017/XrayValidationReportHelp
with specific help available everywhere you see the ⓘ symbol.

The following versions of software and data (see references ⓘ) were used in the production of this report:

MolProbity	:	4.02b-467
Xtriage (Phenix)	:	1.13
EDS	:	trunk30967
Percentile statistics	:	20171227.v01 (using entries in the PDB archive December 27th 2017)
Refmac	:	5.8.0158
CCP4	:	7.0 (Gargrove)
Ideal geometry (proteins)	:	Engh & Huber (2001)
Ideal geometry (DNA, RNA)	:	Parkinson et al. (1996)
Validation Pipeline (wwPDB-VP)	:	trunk30967

1 Overall quality at a glance (i)

The following experimental techniques were used to determine the structure:
X-RAY DIFFRACTION

The reported resolution of this entry is 1.59 Å.

Percentile scores (ranging between 0-100) for global validation metrics of the entry are shown in the following graphic. The table shows the number of entries on which the scores are based.

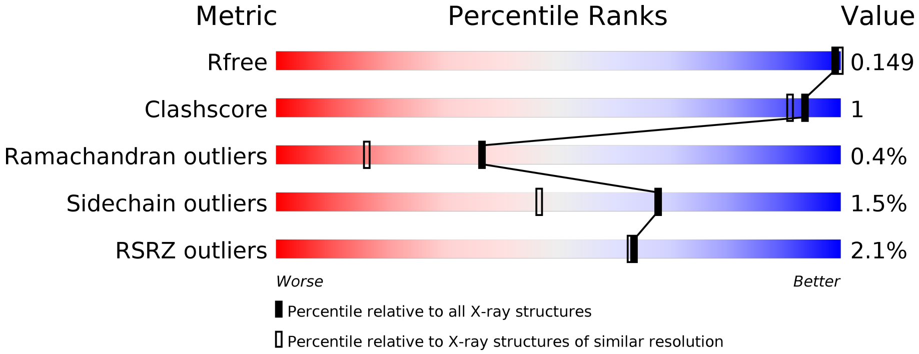

Metric	Whole archive (#Entries)	Similar resolution (#Entries, resolution range(Å))
R_{free}	111664	2957 (1.60-1.60)
Clashscore	122126	3202 (1.60-1.60)
Ramachandran outliers	120053	3117 (1.60-1.60)
Sidechain outliers	120020	3116 (1.60-1.60)
RSRZ outliers	108989	2883 (1.60-1.60)

The table below summarises the geometric issues observed across the polymeric chains and their fit to the electron density. The red, orange, yellow and green segments on the lower bar indicate the fraction of residues that contain outliers for >=3, 2, 1 and 0 types of geometric quality criteria. A grey segment represents the fraction of residues that are not modelled. The numeric value for each fraction is indicated below the corresponding segment, with a dot representing fractions <=5% The upper red bar (where present) indicates the fraction of residues that have poor fit to the electron density. The numeric value is given above the bar.

Mol	Chain	Length	Quality of chain
1	A	185	2% / 92% • •
1	B	185	2% / 92% • •
1	C	185	2% / 91% 5% •
1	D	185	3% / 94% 6%

2 Entry composition (i)

There are 2 unique types of molecules in this entry. The entry contains 12135 atoms, of which 5618 are hydrogens and 0 are deuteriums.

In the tables below, the ZeroOcc column contains the number of atoms modelled with zero occupancy, the AltConf column contains the number of residues with at least one atom in alternate conformation and the Trace column contains the number of residues modelled with at most 2 atoms.

- Molecule 1 is a protein called Ferritin, Dps family protein.

Mol	Chain	Residues	Atoms						ZeroOcc	AltConf	Trace
1	A	179	Total<br>2842	C<br>918	H<br>1397	N<br>244	O<br>280	S<br>3	0	5	0
1	B	179	Total<br>2837	C<br>919	H<br>1390	N<br>244	O<br>281	S<br>3	0	5	0
1	C	179	Total<br>2836	C<br>918	H<br>1391	N<br>244	O<br>280	S<br>3	0	5	0
1	D	185	Total<br>2937	C<br>950	H<br>1440	N<br>254	O<br>289	S<br>4	0	6	0

There are 16 discrepancies between the modelled and reference sequences:

Chain	Residue	Modelled	Actual	Comment	Reference
A	-2	HIS	-	expression tag	UNP B2J981
A	-1	HIS	-	expression tag	UNP B2J981
A	0	PRO	-	expression tag	UNP B2J981
A	2	ALA	SER	engineered mutation	UNP B2J981
B	-2	HIS	-	expression tag	UNP B2J981
B	-1	HIS	-	expression tag	UNP B2J981
B	0	PRO	-	expression tag	UNP B2J981
B	2	ALA	SER	engineered mutation	UNP B2J981
C	-2	HIS	-	expression tag	UNP B2J981
C	-1	HIS	-	expression tag	UNP B2J981
C	0	PRO	-	expression tag	UNP B2J981
C	2	ALA	SER	engineered mutation	UNP B2J981
D	-2	HIS	-	expression tag	UNP B2J981
D	-1	HIS	-	expression tag	UNP B2J981
D	0	PRO	-	expression tag	UNP B2J981
D	2	ALA	SER	engineered mutation	UNP B2J981

- Molecule 2 is water.

Mol	Chain	Residues	Atoms	ZeroOcc	AltConf
2	A	177	Total O<br>177 177	0	0
2	B	165	Total O<br>165 165	0	0
2	C	157	Total O<br>157 157	0	0
2	D	184	Total O<br>184 184	0	0

3 Residue-property plots (i)

These plots are drawn for all protein, RNA and DNA chains in the entry. The first graphic for a chain summarises the proportions of the various outlier classes displayed in the second graphic. The second graphic shows the sequence view annotated by issues in geometryand electron density. Residues are color-coded according to the number of geometric quality criteria for which they contain at least one outlier: green = 0, yellow = 1, orange = 2 and red = 3 or more. A red dot above a residue indicates a poor fit to the electron density (RSRZ > 2). Stretches of 2 or more consecutive residues without any outlier are shown as a green connector. Residues present in the sample, but not in the model, are shown in grey.

- Molecule 1: Ferritin, Dps family protein

Chain A:
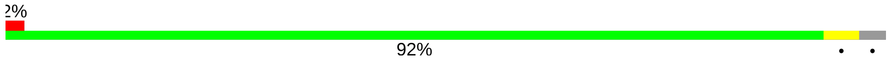

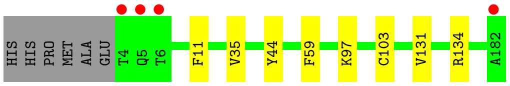

- Molecule 1: Ferritin, Dps family protein

Chain B:
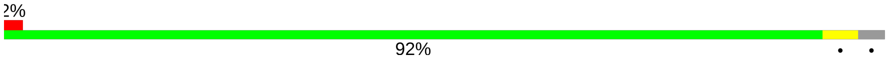

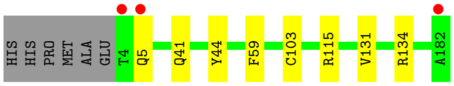

- Molecule 1: Ferritin, Dps family protein

Chain C:
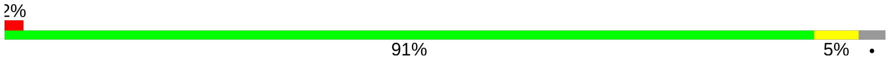

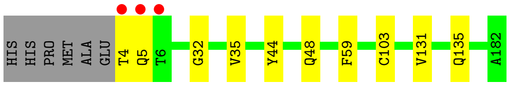

- Molecule 1: Ferritin, Dps family protein

Chain D:
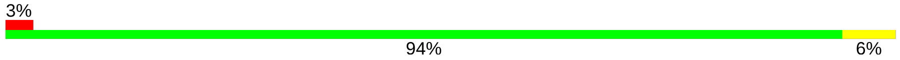

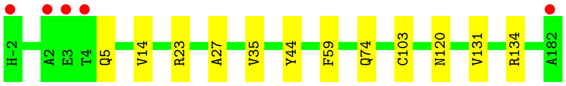

4 Data and refinement statistics (i)

Property	Value	Source
Space group	P 63	Depositor
Cell constants<br>a, b, c, α, β, γ	101.88Å 101.88Å 146.25Å<br>90.00° 90.00° 120.00°	Depositor
Resolution (Å)	48.11 – 1.59<br>48.75 – 1.59	Depositor<br>EDS
% Data completeness<br>(in resolution range)	99.7 (48.11-1.59)<br>97.7 (48.75-1.59)	Depositor<br>EDS
R_{merge}	0.07	Depositor
R_{sym}	(Not available)	Depositor
$< I/\sigma(I) >$ [1]	3.17 (at 1.59Å)	Xtriage
Refinement program	PHENIX	Depositor
R, R_{free}	0.130 , 0.146<br>0.132 , 0.149	Depositor<br>DCC
R_{free} test set	4654 reflections (4.04%)	wwPDB-VP
Wilson B-factor (Å^2)	17.6	Xtriage
Anisotropy	0.104	Xtriage
Bulk solvent k_{sol}(e/Å^3), B_{sol}(Å^2)	0.38 , 42.3	EDS
L-test for twinning[2]	$< \|L\| > = 0.49$, $< L^2 > = 0.32$	Xtriage
Estimated twinning fraction	0.038 for h,-h-k,-l	Xtriage
F_o,F_c correlation	0.98	EDS
Total number of atoms	12135	wwPDB-VP
Average B, all atoms (Å^2)	23.0	wwPDB-VP

Xtriage's analysis on translational NCS is as follows: *The largest off-origin peak in the Patterson function is 3.23% of the height of the origin peak. No significant pseudotranslation is detected.*

[1]Intensities estimated from amplitudes.

[2]Theoretical values of $< |L| >$, $< L^2 >$ for acentric reflections are 0.5, 0.333 respectively for untwinned datasets, and 0.375, 0.2 for perfectly twinned datasets.

5 Model quality (i)

5.1 Standard geometry (i)

The Z score for a bond length (or angle) is the number of standard deviations the observed value is removed from the expected value. A bond length (or angle) with $|Z| > 5$ is considered an outlier worth inspection. RMSZ is the root-mean-square of all Z scores of the bond lengths (or angles).

Mol	Chain	Bond lengths		Bond angles	
		RMSZ	$\#\|Z\| > 5$	RMSZ	$\#\|Z\| > 5$
1	A	0.47	0/1489	0.63	0/2015
1	B	0.46	0/1491	0.63	2/2018 (0.1%)
1	C	0.44	0/1489	0.59	0/2015
1	D	0.47	0/1547	0.61	0/2094
All	All	0.46	0/6016	0.62	2/8142 (0.0%)

There are no bond length outliers.

All (2) bond angle outliers are listed below:

Mol	Chain	Res	Type	Atoms	Z	Observed(^{o})	Ideal(^{o})
1	B	115	ARG	NE-CZ-NH2	6.45	123.52	120.30
1	B	115	ARG	NE-CZ-NH1	-6.07	117.27	120.30

There are no chirality outliers.

There are no planarity outliers.

5.2 Too-close contacts (i)

In the following table, the Non-H and H(model) columns list the number of non-hydrogen atoms and hydrogen atoms in the chain respectively. The H(added) column lists the number of hydrogen atoms added and optimized by MolProbity. The Clashes column lists the number of clashes within the asymmetric unit, whereas Symm-Clashes lists symmetry related clashes.

Mol	Chain	Non-H	H(model)	H(added)	Clashes	Symm-Clashes
1	A	1445	1397	1404	4	0
1	B	1447	1390	1403	3	0
1	C	1445	1391	1404	5	0
1	D	1497	1440	1453	7	0
2	A	177	0	0	0	0
2	B	165	0	0	1	0
2	C	157	0	0	0	0

Continued on next page...

Continued from previous page...

Mol	Chain	Non-H	H(model)	H(added)	Clashes	Symm-Clashes
2	D	184	0	0	1	0
All	All	6517	5618	5664	17	0

The all-atom clashscore is defined as the number of clashes found per 1000 atoms (including hydrogen atoms). The all-atom clashscore for this structure is 1.

All (17) close contacts within the same asymmetric unit are listed below, sorted by their clash magnitude.

Atom-1	Atom-2	Interatomic distance (Å)	Clash overlap (Å)
1:D:23:ARG:NH1	2:D:201:HOH:O	2.12	0.83
1:A:103[B]:CYS:SG	1:A:131:VAL:HG21	2.27	0.75
1:B:103[B]:CYS:SG	1:B:131:VAL:HG21	2.27	0.75
1:C:103[B]:CYS:SG	1:C:131:VAL:HG21	2.29	0.73
1:D:103[B]:CYS:SG	1:D:131:VAL:HG21	2.29	0.71
1:B:103[B]:CYS:SG	2:B:360:HOH:O	2.46	0.67
1:D:35:VAL:CG1	1:D:103[B]:CYS:SG	3.01	0.49
1:D:14:VAL:HG21	1:D:27:ALA:HA	1.95	0.48
1:C:4:THR:N	1:D:120:ASN:HD21	2.11	0.48
1:A:35:VAL:CG1	1:A:103[B]:CYS:SG	3.02	0.47
1:A:131:VAL:HG22	1:A:134:ARG:NH2	2.32	0.45
1:D:131:VAL:HG22	1:D:134:ARG:NH2	2.33	0.44
1:C:32:GLY:HA3	1:C:135:GLN:NE2	2.34	0.43
1:C:35:VAL:CG1	1:C:103[B]:CYS:SG	3.07	0.42
1:C:48:GLN:OE1	1:D:74:GLN:NE2	2.54	0.41
1:A:11:PHE:CE2	1:A:97:LYS:HE2	2.55	0.41
1:B:131:VAL:HG22	1:B:134:ARG:NH2	2.36	0.40

There are no symmetry-related clashes.

5.3 Torsion angles (i)

5.3.1 Protein backbone (i)

In the following table, the Percentiles column shows the percent Ramachandran outliers of the chain as a percentile score with respect to all X-ray entries followed by that with respect to entries of similar resolution.

The Analysed column shows the number of residues for which the backbone conformation was analysed, and the total number of residues.

Mol	Chain	Analysed	Favoured	Allowed	Outliers	Percentiles	
1	A	182/185 (98%)	178 (98%)	4 (2%)	0	100	100
1	B	182/185 (98%)	177 (97%)	4 (2%)	1 (0%)	31	12
1	C	182/185 (98%)	177 (97%)	4 (2%)	1 (0%)	31	12
1	D	189/185 (102%)	184 (97%)	4 (2%)	1 (0%)	31	12
All	All	735/740 (99%)	716 (97%)	16 (2%)	3 (0%)	36	16

All (3) Ramachandran outliers are listed below:

Mol	Chain	Res	Type
1	B	5	GLN
1	C	5	GLN
1	D	5	GLN

5.3.2 Protein sidechains ⓘ

In the following table, the Percentiles column shows the percent sidechain outliers of the chain as a percentile score with respect to all X-ray entries followed by that with respect to entries of similar resolution.

The Analysed column shows the number of residues for which the sidechain conformation was analysed, and the total number of residues.

Mol	Chain	Analysed	Rotameric	Outliers	Percentiles	
1	A	157/157 (100%)	155 (99%)	2 (1%)	71	53
1	B	157/157 (100%)	154 (98%)	3 (2%)	60	36
1	C	157/157 (100%)	155 (99%)	2 (1%)	71	53
1	D	163/157 (104%)	161 (99%)	2 (1%)	74	56
All	All	634/628 (101%)	625 (99%)	9 (1%)	67	50

All (9) residues with a non-rotameric sidechain are listed below:

Mol	Chain	Res	Type
1	A	44	TYR
1	A	59	PHE
1	B	41	GLN
1	B	44	TYR
1	B	59	PHE
1	C	44	TYR
1	C	59	PHE
1	D	44	TYR

Continued on next page...

Continued from previous page...

Mol	Chain	Res	Type
1	D	59	PHE

Some sidechains can be flipped to improve hydrogen bonding and reduce clashes. There are no such sidechains identified.

5.3.3 RNA (i)

There are no RNA molecules in this entry.

5.4 Non-standard residues in protein, DNA, RNA chains (i)

There are no non-standard protein/DNA/RNA residues in this entry.

5.5 Carbohydrates (i)

There are no carbohydrates in this entry.

5.6 Ligand geometry (i)

There are no ligands in this entry.

5.7 Other polymers (i)

There are no such residues in this entry.

5.8 Polymer linkage issues (i)

There are no chain breaks in this entry.

6 Fit of model and data ⓘ

6.1 Protein, DNA and RNA chains ⓘ

In the following table, the column labelled '#RSRZ> 2' contains the number (and percentage) of RSRZ outliers, followed by percent RSRZ outliers for the chain as percentile scores relative to all X-ray entries and entries of similar resolution. The OWAB column contains the minimum, median, 95th percentile and maximum values of the occupancy-weighted average B-factor per residue. The column labelled 'Q< 0.9' lists the number of (and percentage) of residues with an average occupancy less than 0.9.

Mol	Chain	Analysed	<RSRZ>	#RSRZ>2			OWAB(Å^2)	Q<0.9
1	A	179/185 (96%)	-0.55	4 (2%)	62	61	14, 18, 31, 59	1 (0%)
1	B	179/185 (96%)	-0.55	3 (1%)	70	69	14, 18, 31, 72	1 (0%)
1	C	179/185 (96%)	-0.58	3 (1%)	70	69	14, 18, 32, 58	0
1	D	185/185 (100%)	-0.48	5 (2%)	54	53	14, 17, 36, 89	0
All	All	722/740 (97%)	-0.54	15 (2%)	63	63	14, 18, 33, 89	2 (0%)

All (15) RSRZ outliers are listed below:

Mol	Chain	Res	Type	RSRZ
1	D	4	THR	9.5
1	B	4	THR	5.8
1	D	-2	HIS	5.7
1	C	4	THR	5.3
1	A	4	THR	5.1
1	A	182	ALA	4.1
1	B	5	GLN	3.8
1	D	3	GLU	3.8
1	A	5	GLN	3.7
1	C	5	GLN	3.5
1	B	182	ALA	2.7
1	A	6	THR	2.3
1	D	182	ALA	2.2
1	D	2	ALA	2.1
1	C	6	THR	2.1

6.2 Non-standard residues in protein, DNA, RNA chains ⓘ

There are no non-standard protein/DNA/RNA residues in this entry.

6.3 Carbohydrates ⓘ

There are no carbohydrates in this entry.

6.4 Ligands ⓘ

There are no ligands in this entry.

6.5 Other polymers ⓘ

There are no such residues in this entry.
